# Editorial: Precision Medicine in Oncology

**DOI:** 10.3389/fonc.2018.00479

**Published:** 2018-10-26

**Authors:** Angela Re, Caterina Nardella, Alessandro Quattrone, Andrea Lunardi

**Affiliations:** ^1^Systems and Synthetic Biology Laboratory, Centre for Sustainable Future Technologies, Fondazione Istituto Italiano di Tecnologia, Torino, Italy; ^2^Precision Medicine Initiative, Centre for Integrative Biology, Trento, Italy; ^3^Translational Genomics Laboratory, Centre for Integrative Biology, Trento, Italy; ^4^Armenise-Harvard Cancer Biology & Genetics Laboratory, Centre for Integrative Biology, Trento, Italy

**Keywords:** precision medicine, oncology, pathology, *in vivo* preclinical platform, computational biology, non-coding RNA

Recent advances in technology have unveiled a tremendous heterogeneity in cancer dysfunctional mechanisms. This gain of knowledge has opened a new era in oncology, which relays on the concept that each tumor is different and should be treated in a specific way depending on its distinctive molecular dysfunctions. Fundamental achievements in cancer biology paralleled by unprecedented improvements in disease modeling from all *in silico, in vitro* and *in vivo* perspectives, have converged to offer nowadays the compelling opportunity to design therapeutic approaches tailored on individual patients, namely precision medicine.

This Research Topic embodies 13 multidisciplinary manuscripts focused on multifaceted aspects related to “Precision Medicine in Oncology.” Overall, each investigator discusses some of the numerous pending issues associated with this ground-breaking field, ranging from basic research findings, novel technologies, and computational approaches to potential innovative translational venues and widely needed new platforms for precision medicine implementation. Specifically, this issue includes: (i) original research reports on novel biological findings and an innovative technology for immunotherapy; (ii) comprehensive reviews on key cancer biomarkers, signaling, and metabolic pathways as well as on theoretical and preclinical models, and analytical integrative methodologies; (iii) insightful perspectives on advanced computational platforms as well as on a novel integrated murine/human clinical infrastructure.

A key aspect for accelerating the development of new effective targeted therapies is represented by a deeper, faster and broader genomic characterization of patient samples. The National Cancer Institute is currently leading numerous multi-disciplinary projects aiming at facilitating the development of precision oncology diagnostics and therapeutic treatments. In a timely review hosted in this Research Topic, Hinkson et al. introduce the Genomic Data Commons (GDC) initiative, which redistributes high quality data and metadata and three Cloud Resources, thus supporting cloud-based access to data, computational scalability and collaboration. Additionally, the review from Davis' group provides an insightful overview on catalogs, software and tools useful for the interpretation of single nucleotide variants and short insertions and deletions in point-of-care high throughput sequencing applications (Tsang et al).

Deep genome and transcriptome sequencing are having two major roles in: (i) facilitating the discovery of new pathways and molecular players involved in cancer onset, progression and drug resistance, thereby offering the opportunity to identify more reliable biomarkers and novel druggable targets; (ii) revolutionizing the clinical approach to human diseases as a result of the unprecedented characterization of the non-coding space of our genome.

While the non-coding dark matter still remains a challenging target *in vivo*, pharmacological tuning of the coding space has been shown to yield promising results *in vitro* and, to a certain extent, in preclinical and clinical trials. Comunanza and Bussolino describe the insights gained on the vascular-endothelial growth factor (VEGF) over the last 40 years and relevant challenges raised by VEGF-targeted anti-angiogenic therapies (Comunanza and Bussolino). They deeply review the emergence of approaches combining anti-angiogenic regimens with compounds targeting angiogenic mechanisms, oncogenic drivers, and immunotherapy (Comunanza and Bussolino).

In an original research article, Astrologo and colleagues provide evidence that the Bone Morphogenetic Protein 9 (BMP9) might represent a novel therapeutic target in prostate cancer (Astrologo et al). They nicely demonstrate that preventing BMP9 binding to its cell surface receptors, and thus blocking BMP9 signaling, efficiently diminishes prostate cancer cell proliferation and substantially attenuates tumor growth in both an orthotopic model of human prostate cancer and a xenograft derived from an androgen-dependent bone metastatic prostate tumor patient (Astrologo et al).

In respect of non-coding elements, Montironi's group mini review focused on *in vitro* and *in vivo* gain-of-function and loss-of-function experiments showing that long non-coding RNAs play a crucial role in cancer cell invasiveness and metastasis through antagonizing the genome-wide localization and regulatory functions of the SWI/SNF chromatin-modifying complex (Cimadamore et al.) In addition to long non-coding RNAs, another class of regulatory RNAs, namely microRNAs, have been implicated in nearly every signaling pathway. Specifically, microRNA-mediated altered signaling pathway regulation appears to affect a heterogeneous spectrum of cancer behaviors. In this respect, Denti's group provide a comprehensive overview of the tight connection between microRNA misfunction and cancer hallmarks (Detassis et al). They also thoroughly discuss benefits and hurdles of microRNAs as biomarkers to move personalized cancer biogenesis, evolution, diagnosis, and treatment a step forward. Additionally, Gabra and Salmena's review contributes to the debate on the role of microRNAs in personalized cancer therapy focusing on drug resistance and the mechanisms of action that lead to poor overall survival. They also discuss the potential clinical use of miRNA mimic- or antagomir-based approaches in drug resistance overcome (Gabra and Salmena).

Interestingly, two contributors pointed out to the relevance of approaches encompassing metabolism to develop suitable cancer-specific treatments. The extensive crosstalk within and between reactive oxygen species (ROS) detoxification, redox signaling transduction, energy metabolism and central metabolism has been finely reviewed in this Research Topic by Benfeitas et al. The outlined reconstruction of redox metabolism has been connected to the heterogeneity in redox responses displayed by different types of cancer, between individuals affected by the same form of tumor, as well as within different cancer stages. They also highlighted the utility of system-level approaches to capture the role of redox systems in cancer and to design redox-targeting drugs producing synergistic responses for cancer treatment or prevention (Benfeitas et al). On another review, Martín-Martín and colleagues accurately depict the complex interrelationship between metabolism and gene expression regulation in cancer (Martín-Martín et al). The authors report recent advances highlighting how the tight and dynamic coordination between gene expression programs and metabolism dictates cellular adaptations during cancer progression and might lead to new therapeutic opportunities (Martín-Martín et al).

Although counteracting pro-tumorigenic stimuli has always been a major goal in oncology, alternative innovative therapeutic strategies are currently emerging impetuously. Among the most promising ones, we highlight here the synthetic lethality approach and cancer vaccines.

As reviewed by Caffo's group, impairment of DNA damage repair pathways is a common event in cancer, resulting in genomic instability which is crucial for the tumorigenic process (Caffo et al). Exacerbation of such a condition through the administration of DNA damage agents in combination with molecules further affecting DNA repair pathways has been shown to effectively result in cancer cell death (Caffo et al). Importantly, Caffo and colleagues discuss the relevance of applying DNA sequencing approaches for the screening of genomic aberrations affecting DNA repair pathways in prostate cancer with the ultimate goal of stratifying prostate cancer patients for personalized synthetic lethal therapeutic approaches (Caffo et al).

In an original research article, Grandi's group explore the applicability of the Outer Membrane Vesicles (OMVs) platform technology in cancer vaccination (Grandi et al). Technological promising aspects of OMVs, such as the rapidity they can be decorated with foreign epitopes, the high yield production from bacterial fermentation and the easy purification process, inspired the authors to test OMVs amenability for cancer vaccines. Immunization with OMVs engineered with the B cell cancer-specific epitope strongly protected mice from tumor development once injected with a syngeneic cancer cell line expressing the epitope on its surface (Grandi et al). Finally, the synergistic protective activity of multiple epitopes administered with OMVs was found to potentiate the overall efficacy of the OMV cancer vaccine (Grandi et al).

From a clinical perspective, our deeper understanding of oncogenic mechanisms has recently begun to have a crucial impact on clinical decisions at several steps, from cancer prevention and diagnosis to therapeutic intervention. Nowadays, the development of innovative investigational *in silico, in vitro*, and *in vivo* platforms fostering the clinical translational potential of basic research findings is of primary relevance.

In this Research Topic, Re reviews significant advancements in our capabilities to tailor synthetic genetic circuits to specific applications in tumor diagnosis, tumor cell- and gene-based therapy, and drug delivery (Re). From a different perspective, Clohessy and Pandolfi present the Mouse Hospital and the Co-Clinical Trial Project focused on the integration of data collected from cancer patients and faithful cancer mouse models enrolled in concomitant trials (co-clinical trials) with identical treatment protocols. They discuss how co-clinical studies can quickly lead to effective clinical decisions by predicting patients' drug response on genetic and molecular bases as well as by anticipating effective second line treatments for drug resistance-driven cancer relapse (Clohessy et al).

Altogether, the original articles, reviews and perspectives collected in this Research Topic represent an invaluable resource of insights on important achievements attained so far in identifying altered molecular events that lead to the development of cancer and therapy resistance as well as novel therapeutic strategies for the successful delivery of precision medicine approaches in oncology.

## Author contributions

All authors listed have made a substantial, direct and intellectual contribution to the work, and approved it for publication.

### Conflict of interest statement

The authors declare that the research was conducted in the absence of any commercial or financial relationships that could be construed as a potential conflict of interest.

